# Radiation therapy compared to radical prostatectomy as first-line definitive therapy for patients with high-risk localised prostate cancer: An updated systematic review and meta-analysis

**DOI:** 10.1080/2090598X.2022.2026010

**Published:** 2022-03-30

**Authors:** Abdulmajeed Aydh, Reza Sari Motlagh, Mohammad Abufaraj, Keiichiro Mori, Satoshi Katayama, Nico Grossmann, Pawel Rajwa, Hadi Mostafai, Ekaterina Laukhtina, Benjamin Pradere, Fahad Quhal, Victor M. Schuettfort, Alberto Briganti, Pierre I. Karakiewicz, Haron Fajkovic, Shahrokh F. Shariat

**Affiliations:** a Department of Urology, Comprehensive Cancer Center, Medical University of Vienna, Vienna, Austria; b Department of Urology, King Faisal Medical City, Abha, Saudi Arabia; c Men’s Health and Reproductive Health Research Center, Shahid Beheshti University of Medical Sciences, Tehran, Iran; d The National Center for Diabetes, Endocrinology and Genetics, The University of Jordan, Amman, Jordan; e Department of Urology, The Jikei University School of Medicine, Tokyo, Japan; f Department of Urology, Okayama University Graduate School of Medicine, Dentistry and Pharmaceutical Sciences, Okayama, Japan; g Department of Urology, University Hospital Zurich, Zurich, Switzerland; h Department of Urology, Medical University of Silesia, Zabrze, Poland; i Research Center for Evidence Based Medicine, Tabriz University of Medical Sciences, Tabriz, Iran; j Institute for Urology and Reproductive Health, Sechenov University, Moscow, Russia; k Department of Urology, University Hospital of Tours, Tours, France; l Department of Urology, King Fahad Specialist Hospital, Dammam, Saudi Arabia; m Department of Urology, University Medical Center Hamburg-Eppendorf, Hamburg, Germany; n Division of Oncology/Unit of Urology, URI, IRCCS Ospedale San Raffaele, Milan, Italy; o Cancer Prognostics and Health Outcomes Unit, University of Montreal Health Center, Montreal, Canada; p Department of Urology, Weill Cornell Medical College, New York, NY, USA; q Department of Urology, University of Texas Southwestern, Dallas, TX, USA; r Department of Urology, Second Faculty of Medicine, Charles University, Prague, Czech Republic; s Karl Landsteiner Institute of Urology and Andrology, Vienna, Austria; t Hourani Center for Applied Scientific Research, Al-Ahliyya Amman University, Amman, Jordan

**Keywords:** Brachytherapy, external beam radiation therapy, high-risk prostate cancer, radical prostatectomy, radiation therapy, androgen-deprivation therapy

## Abstract

**Objective:**

To present an update of the available literature on external beam radiation therapy (EBRT) with or without brachytherapy (BT) compared to radical prostatectomy (RP) for patients with high-risk localised prostate cancer (PCa).

**Methods:**

We conducted a systematic review and meta-analysis of the literature assessing the survival outcomes in patients with high-risk PCa who received EBRT with or without BT compared to RP as the first-line therapy with curative intent. We queried PubMed and Web of Science database in January 2021. Moreover, we used random or fixed-effects meta-analytical models in the presence or absence of heterogeneity per the *I^2^* statistic, respectively. We performed six meta-analyses for overall survival (OS) and cancer-specific survival (CSS).

**Results:**

A total of 27 studies were selected with 23 studies being eligible for both OS and CSS. EBRT alone had a significantly worse OS and CSS compared to RP (hazard ratio [HR] 1.38, 95% confidence interval [CI] 1.16–1.65; and HR 1.55, 95% CI 1.25–1.93). However, there was no difference in OS (HR 1.1, 95% CI 0.76–1.34) and CSS (HR 0.69, 95% CI 0.45–1.06) between EBRT plus BT compared to RP.

**Conclusion:**

While cancer control affected by EBRT alone seems inferior to RP in patients with high-risk PCa, BT additive to EBRT was not different from RP. These data support the need for BT in addition to EBRT as part of multimodal RT for high-risk PCa.

**Abbreviations:** ADT: androgen-deprivation therapy; BT: brachytherapy; CSS: cancer-specific survival; HR: hazard ratio; MFS, metastatic-free survival; MOOSE: Meta-analyses of Observational Studies in Epidemiology; OR: odds ratio; OS: overall survival; PCa: prostate cancer; RR: relative risk; RP: radical prostatectomy; RCT: randomised controlled trials; (EB)RT: (external beam) radiation therapy

## Introduction

High-risk non-metastatic disease (i.e. PSA level >20 ng/mL, Gleason score of 8, and/or a clinical stage of T2c–3a) accounts for ~30% of newly diagnosed prostate cancer (PCa) [1,2], depending on its definition [3,4]. The optimal primary treatment for these patients remains unresolved with standard local therapeutic options including radical prostatectomy (RP) and radiation therapy (RT) consisting of external beam RT (EBRT) with or without brachytherapy (BT). Owing to the heterogeneous nature of the published cohort studies and the lack of prospective randomised controlled trials (RCTs), it remains unclear which single and/or multimodal therapeutic strategy is optimal for each patient with high-risk PCa.

In the current and likely future, the absence of propensity designed RCTs comparing RP to RT for patients with high-risk PCa, meta-analyses may help a framework for clinical decision-making and patient counselling. To this end, we performed a systematic review and meta-analysis to summarise the results of available studies including the latest literature on this subject. We focussed on the additive value of BT in addition to EBRT.

## Methods

In this meta-analysis, we followed the Meta-analyses of Observational Studies in Epidemiology (MOOSE) statement guidelines that propose a checklist of items which provides a RCTs checklist [5]. Moreover, the Preferred Reporting Items for Systematic Reviews and Meta-Analysis (PRISMA) was used.

### Eligibility criteria

The question of this study was, ‘Do patients with high-risk PCa who receive EBRT with or without BT have a better survival outcome compared to those who receive RP?’. All current articles covering the study question were eligible for this systematic review. We selected studies that perform quantitative synthesis according to the similarity in Population, Interest, Context (PICO) elements to decrease the selection bias and heterogeneity. The inclusion criteria for the quantitative meta-analysis were original research articles that assessed survival outcomes and reported an estimated risk effect (hazard ratio [HR], odds ratio [OR], relative risk [RR]) for both patient and control groups. Exclusion criteria were BT usage alone as definitive therapy and lack of definition of high-risk PCa in the main or subgroup analysis.

Consequently, the more comparable cohort studies according to the MOOSE guidelines were included in the analyses. Furthermore, the heterogeneity of the population was explored by detecting the source and country of databases. According to OS, we categorised studies’ outcomes, CSS, biochemical recurrence survival, and metastatic-free survival (MFS).

### Information sources

We searched PubMed and the Web of Science for studies published before 1 January 2021. The search queries line and search strategies were ‘((Prostate OR prostatic) AND (cancer OR carcinoma) OR (Prostatic Neoplasms[Mesh])) AND external beam radiotherapy OR brachytherapy OR radiotherapy [Mesh]) AND (radical prostatectomy [Mesh])’ in PubMed and ‘((Prostate OR prostatic) AND (cancer OR carcinoma)) AND (external beam radiotherapy OR brachytherapy OR radiotherapy) AND (radical prostatectomy)’ in the Web of Science.

The search results were restricted to English language articles. Two reviewers screened titles and abstracts independently; any disagreement about the articles’ eligibility was resolved by Delphi consensus with the co-authors. A data extraction sheet was developed based on the Cochrane Consumers and the Communication Review Group’s (http://cccrg.cochrane.org/author-resources). We extracted the following data: first-author, type of article, year of publication, dates of the data collection or enrolment, cohort type, sample size, number of individuals on treatment, outcome, how the outcome was measured, type of effect statistic, effect statistic error measures, and effect statistic *P* value. There were no limitations in the articles’ data, so we did not need to contact any authors for additional details. Modified Newcastle-Ottawa Scale criteria were used to assess the quality of the included studies [6]. Subsequently, the HRs and 95% CIs with OS, CSS outcomes were retrieved, all discrepancies regarding data extraction were resolved by Delphi consensus with co-authors.

### Statistical analysis

Forest plots were used to assess the multivariable HRs. We summarised them to depict the relationship of our outcomes with the type of treatment (i.e. RP, EBRT alone and EBRT plus BT). When HRs and *P* value only were reported, we calculated the corresponding 95% CIs. We utilised multivariable adjusted or propensity score matched analyses in the quantitative meta-analyses. Studies included in performing the meta-analyses were adjusted for the effects of age, clinical T stage, Gleason grade, and PSA. The primary meta-analysis was performed for all studies that reported OS as an outcome. A secondary meta-analysis was conducted using studies that reported CSS as an outcome. The next four meta-analyses were conducted among studies that reported the risk of OS and CSS according to the type of RT (i.e. EBRT alone or EBRT plus BT). Heterogeneity across the studies was appraised using *P* values, Q and *I^2^* statistics [7]. In the presence of statistically significant heterogeneity (˃50%), random effect meta-analysis was used. When there was no significant heterogeneity observed, the fixed-effect model was used. Funnel plots was used to detect the risk of publication bias. Statistical analyses were considered significant if the *P* value was <0.05. All analyses were carried out using Stata version 14 (Stata Corp., College Station, TX, USA).

## Results

**Figure 1.**
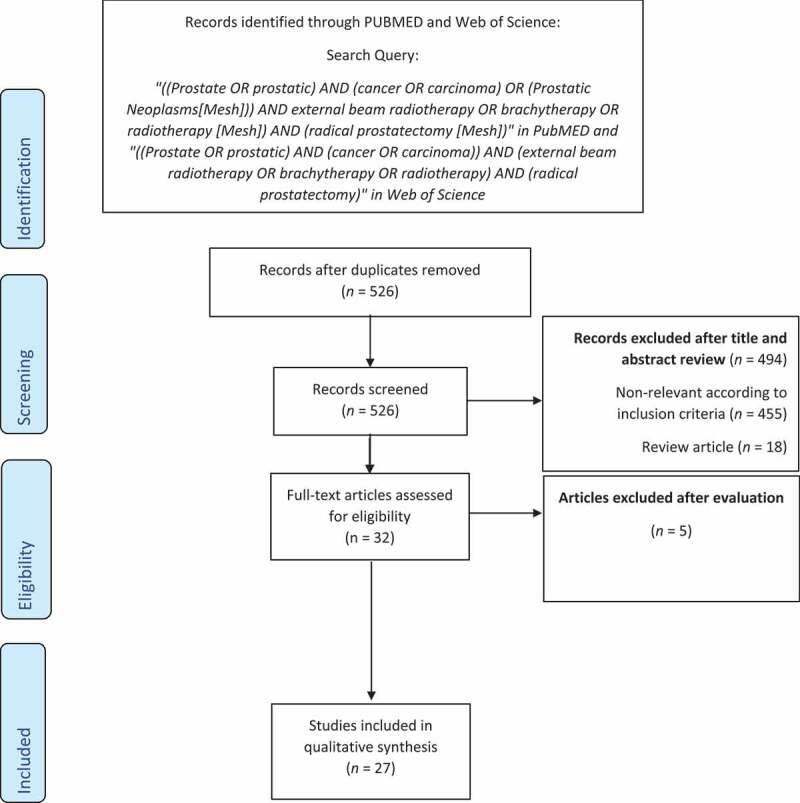
The selection process of the articles to assess survival outcomes among patients with high-risk prostate cancer who received RT compared to RP.

After initial screening, 526 articles were available for assessment. The selection process for the systematic review is shown in Figure 1. With further assessment, according to inclusion and exclusion criteria, 27 studies were finally available for the systematic review and meta-analysis (Table 1) [8–34].

**Table 1.** Characteristics of the included studies of patients treated locally for high-risk prostate cancer

Study	Year	High-risk PCa definition	Sample size, *n*	Intervention group	Control group	Outcomes	Radiation dose
Yasui et al. Japan (1970–2014) [8]	2020	T3	4810	EBRT	RP+ sRT	OS/CSS	NR
Zhou et al. [9]	2020	T3	9258	EBRT EBRT+BT	RP	OS/CSS	NR
Muralidhar et al. NCDB and SEER (2004–2012) [10]	2019 NCBD/SEER	GS: 9–10	4367/2278	EBRT+BT	RP+aRT	OS	NR
Knipper et al. SEER 2004–2015 [11]	2019	GS: 9–10	16,018	EBRT	RP+aRT	OS/CSS	NR
Yin et al. SEER (2004–2015) [12]	2019	NCCN	62178	EBRT EBRT+BT	RP+aRT	OS/CSS	NR
Berg et al. NCDB (2004–2009) [13]	2019	NCCN	13985	EBRT+BT	RP	OS	NR
Jayadevappa et al. SEER–Medicare (1996–2003) [14]	2019	GS: ≥8 or T≥ T2c	4878	EBRT EBRT + BT	RP	OS/CSS	NR
Reichard et al. MD Anderson (2004–2013); comparison with matched SEER cohort [15]	2019	NCCN	304	EBRT	RP	OS/MFS	75.6 Gy
Ennis et al. NCDB (2004–2013) [16]	2018	NCCN	40123	EBRT EBRT+BT	RP	OS	NR
Tilki et al. Chicago Prostate Cancer Center, USA, and Martini-Klinik Prostate Cancer Center, Germany (1992–2013) [17]	2018	GS: 9–10	452	EBRT+BT	RP+aRT	CSS/OS	45 Gy IMRT + BT: ^125^I (108 Gy), ^103^Pa (90 Gy), ^131^Cs (100 Gy)
Jang et al. SEER Medicare (1992–2009) [18]	2018	≥T3a or GS 8–10	7946	EBRT	RP+aRT	CSS/OS	NR
Kishan et al. University of California, Los Angeles (2000–2013) [19]	2018	NCCN	1373	EBRT EBRT+BT	RP	CSS/OS/ MFS	XRT: median 74.3 Gy XRT+BT: median 91.5 Gy
Robinson et al. NPCR of Sweden (1998–2012) [20]	2018	NCCN	41503	EBRT	RP	CSS	
Markovina et al. Washington University, St. Louis (2002–2011) [21]	2018	NCCN	124	EBRT	RP	OS/MFS	Median 75.6 Gy
Gu et al. SEER (2004–2008) [22]	2018	NCCN	7656	EBRT	RP	CSS/OS	NR
Feldman et al. SEER–Medicare (1992–2009) [23]	2017	T3	2935	EBRT	RP	OS/CSS	NR
Ciezki et al. Cleveland Clinic (1996–2012) [24]	2016	NCCN	2042	EBRT	RP	CSS/BRFS	78 Gy
Taguchi et al. University of Tokyo (2005–2012) [34]	2015	D’Amico	336	EBRT	RP	OS/CSS/BRFS	Median 76 Gy
Yamamoto et al. Japan (1994–2005) [25]	2014	T3	231	EBRT	RP	OS/CSS	70 Gy (60–72)
Sooriakumaran et al. PCBaSe Sweden (1996–2010) [33]	2014	Modified NCCN	7649	EBRT	RP	CSS	NR
Merino et al. Pontificia Universidad Catolica de Chile (1999–2010) [26]	2013	D’Amico	294	EBRT	RP	CSS/BRFS	76 Gy
Hoffman et al. PCOS (1994–2010) [27]	2013	PSA >10 ng/mL or GS 8–10	437	EBRT	RP	OS/CSS	NR
Sun et al. SEER (1992–2005) [28]	2013	T2c	5945	EBRT	RP	OS/CSS	NR
Westover et al. 21st century oncology, Chicago Prostate Center, Duke University (1988–2008) [29]	2012	D’Amico	657	EBRT+BT	RP	CSS	45 Gy RT+ 90–108 Gy BT
Kibel et al. Barnes-Jewish Hospital and Cleveland Clinic (1995–2005) [30]	2012	D’Amico	1201	EBRT	RP	OS/CSS	Median 74 Gy (Barnes Jewish) and 78 Gy (Cleveland Clinic)
Abdollah et al. SEER (1992–2005) [31]	2012	T2c or GS 8–10	6057	EBRT	RP	OS/CSS	NR
Boorjian et al. MayoClinic, Fox Chase (1988–2004) [32]	2011	NCCN	1582	EBRT	RP	OS/CSS	72 Gy (50–79)

aRT: adjuvant RT; BRFS: biochemical recurrence-free survival; GS: Gleason score; IMRT: intensity modulated RT; NCDB: National Cancer Database; NCCN: National Comprehensive Cancer Network; NR: not reported; PCBaSe: Prostate Cancer data Base Sweden; PCOS: Prostate Cancer Outcomes Study; SEER: Surveillance, Epidemiology and End Results; sRT: salvage radiotherapy.

Almost all of the studies in this review were cohort studies. Of the 27 included studies, regardless of the type of RT (i.e. EBRT alone or EBRT plus BT), 23 studies assessed OS and CSS. The quality assessment of the included studies according to the Newcastle-Ottawa scale is summarised in Table 2 [8–34]. In general, there were only two fair quality studies, while all 25 others had at least good quality (Table 2).

**Table 2.** The Newcastle-Ottawa scale for all studies in the quantitative synthesis

Study	Selection	Comparability	Outcome	Total
Yasui et al. [8]	****	**	**	8
Zhou et al. [9]	****	**	**	8
Muralidhar et al. [10]	****	**	**	8
Knipper et al. [11]	****	**	***	9
Yin et al. [12]	****	**	**	8
Berg et al. [13]	****	**	**	8
Jayadevappa et al. [14]	****	**	**	8
Reichard et al. [15]	****	**	**	8
Ennis et al. [16]	****	**	*	7
Tilki et al. [17]	***	**	*	6
Jang et al. [18]	****	**	**	8
Kishan et al. [19]	****	**	*	7
Robinson et al. [20]	****	**	*	7
Markovina et al. [21]	****	**	*	7
Gu et al. [22]	****	**	**	8
Feldman et al. [23]	****	**	**	8
Ciezki et al. [24]	****	**	**	8
Taguchi et al. [34]	****	**	*	7
Yamamoto et al. [25]	****	**	**	8
Sooriakumaran et al. [33]	****	**	***	9
Merino et al. [26]	****	**	*	7
Hoffman et al. [27]	****	**	***	9
Sun et al. [28]	****	**	*	7
Westover et al. [29]	***	**	*	6
Kibel et al. [30]	****	**	**	8
Abdollah et al. [31]	****	**	**	8
Boorjian et al. [32]	***	**	**	7

*According to Newcastle-Ottawa scale, stars were awarded for each quality item such that highest quality studies were awarded up to 9 stars.

### OS and CSS for patients with high-risk PCa who received RT compared to RP

**Figure 2.**
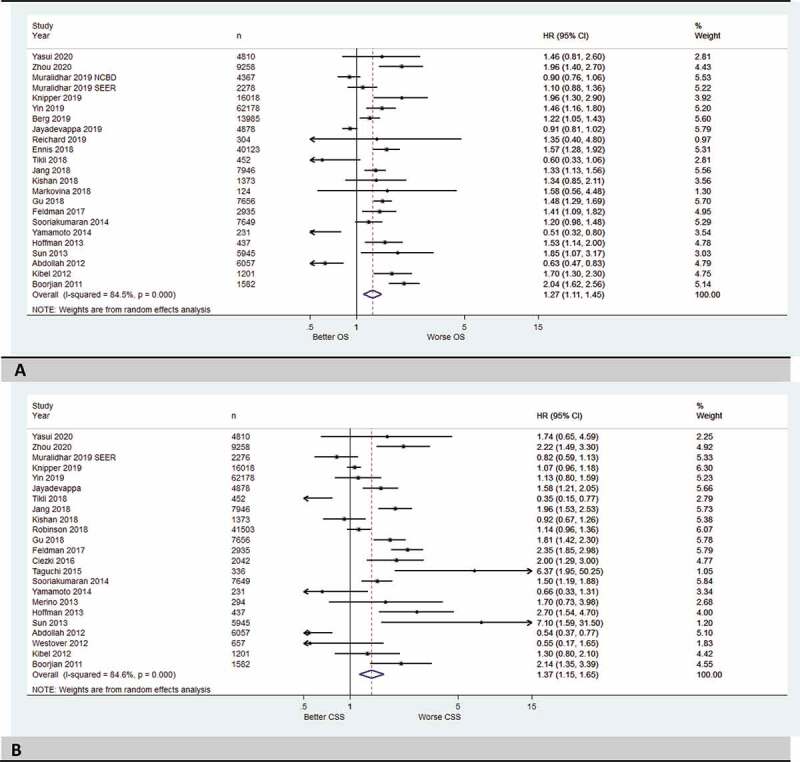
(a) Forest plots with summary HRs (all included study) for OS of the RT group vs RP (reference group) for high-risk prostate cancer. (b) Forest plots with summary HRs (all included study) for CSS of the RT group vs RP (reference group) for high-risk prostate cancer.

In the first meta-analysis of OS, 23 were included. We found that patients with high-risk PCa who received RT (regardless of the type of RT) had significantly worse OS than those treated with RP with a HR of 1.27 (95% CI 1.11–1.45; Figure 2(a)). The 23 studies included in the meta-analysis demonstrated a high heterogeneity (*I*^2^ = 84.5%, *P < *0.001), so a random-effect model was used. The funnel plot was asymmetrical (Figure1S-A in supplemental data). In the second meta-analysis, 23 studies were again included. We found that patients with high-risk PCa who received RT (regardless of the type of RT) had significantly worse CSS than those treated with RP with a HR of 1.37 (95% CI 1.15–1.65; Figure 2(b)). The 23 studies included in the meta-analysis showed a high heterogeneity (*I*^2^ = 84.6%, *P < *0.001), so a random-effect model was used. The funnel plot was asymmetrical (Figure1S-B in supplemental data).

### OS and CSS for patients with high-risk PCa who received only EBRT compared to RP

**Figure 3.**
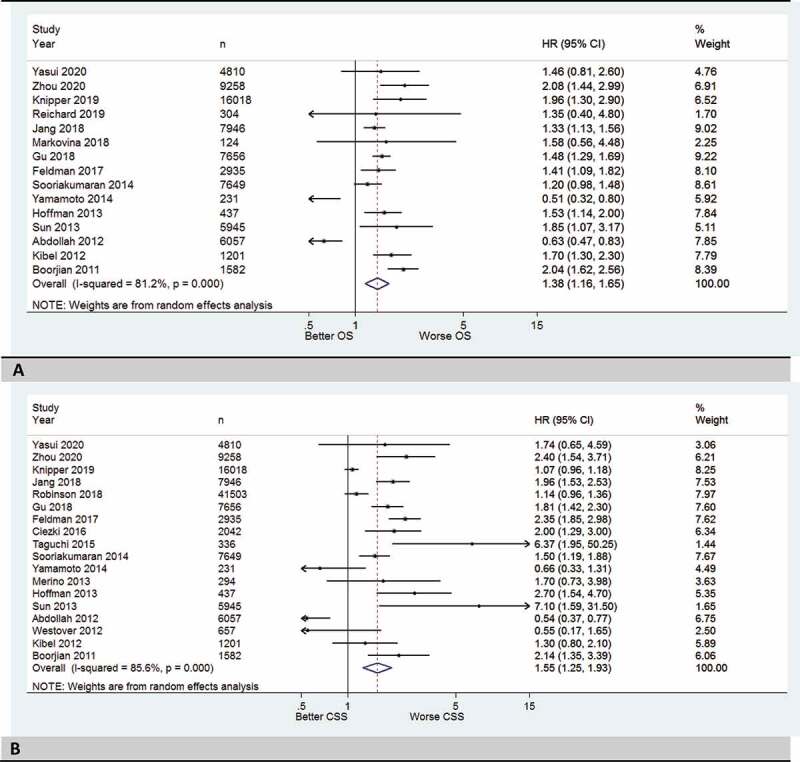
(a) Forest plots with summary HRs (only EBRT) for OS of the RT group vs RP (reference group) for high-risk prostate cancer. (b) Forest plots with summary HRs (only EBRT) for CSS of the RT group vs RP (reference group) for high-risk prostate cancer.

In the first subgroup meta-analysis, 15 studies were included. We found that patients with high-risk PCa who received EBRT alone (i.e. without a concomitant BT) had significantly worse OS than those treated with RP with a HR of 1.38 (95% CI 1.16–1.65; Figure 3(a)). The 15 studies included in the meta-analysis demonstrated a high heterogeneity (*I*^2^ = 81.2%, *P < *0.001), so a random-effect model was used. The funnel plot was slightly asymmetrical (Figure1S-C in supplemental data). In the second subgroup meta-analysis, 18 studies were included. We found that patients with high-risk PCa who received EBRT alone had significantly worse CSS than those treated with RP with a HR of 1.55 (95% CI 1.25–1.93; Figure 3(b)). The 18 studies included in the meta-analysis showed a high heterogeneity (*I*^2^ = 85.6%, *P < *0.001), so a random-effect model was used. The funnel plot was slightly asymmetrical (Figure1S-D in supplemental data).

### OS and CSS for patients with high-risk PCa who received EBRT plus BT compared to RP

**Figure 4.**
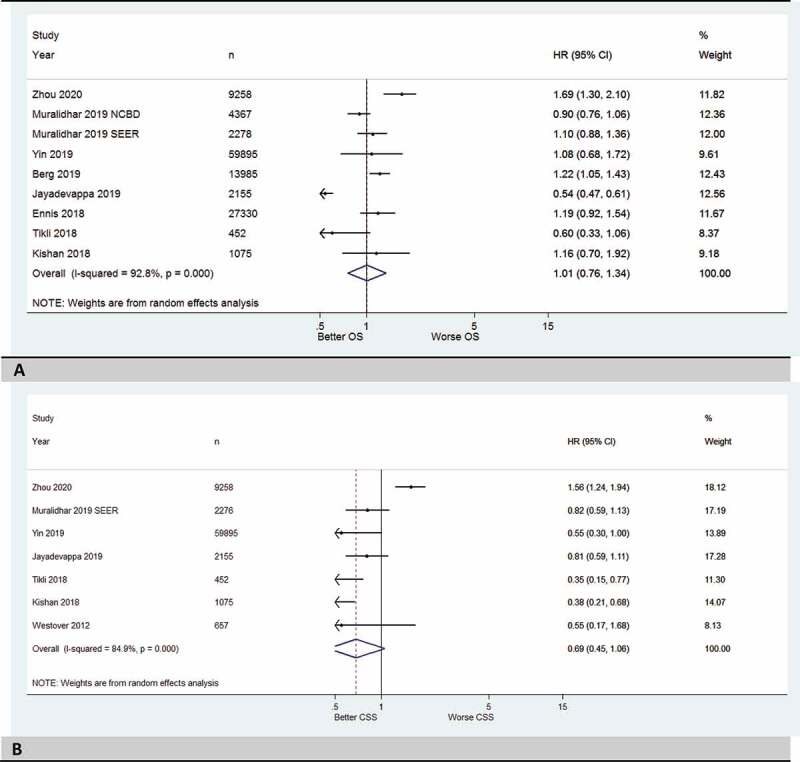
(a) Forest plots with summary HRs (EBRT plus BT) for OS of the RT group vs RP (reference group) for high-risk prostate cancer. (b) Forest plots with summary HRs (EBRT plus BT) for CSS of the RT group vs RP (reference group) for high-risk prostate cancer.

In the third subgroup meta-analysis, eight studies were included. The HR of 1.1 (95% CI 0.76–1.34) suggested that there was no difference in OS between patients with high-risk PCa who received EBRT plus BT compared to those who underwent RP (Figure 4(a)). The eight studies included in the meta-analysis demonstrated a high heterogeneity (*I*^2^ = 92.8%, *P < *0.001), so a random-effect model was used. The funnel plot was asymmetrical (Figure1S-E in supplemental data). In the fourth subgroup meta-analysis, seven studies were included. The HR of 0.69 (95% CI 0.45–1.06) suggested no difference in CSS between patients with high-risk PCa who received EBRT plus BT and those who underwent RP; however, statistical significance was not reached (Figure 4(b)). The seven studies included in the meta-analysis showed moderate heterogeneity (*I*^2^ = 84.9%, *P* = 0.096), so a random-effect model was used. The funnel plot was slightly asymmetrical (Figure1S-F in supplemental data). Table 3 [9,10,12–14,16,17,19,29] shows the reported data regarding RT and RP components among studies included in this systematic review.

**Table 3.** Reported data regarding multimodal therapy among studies that used EBRT plus BT compared to RP

Study	Adjuvant ADT, %		Adjuvant or salvage RT, %
	EBRT plus BT	RP	RP
Zhou et al. [9]	NR	NR	NR
Muralidhar et al. [10]	NR	NR	NR
Yin et al. [12]	NR	NR	NR
Berg et al. [13]	69	15	15
Jayadevappa et al. [14]	NR	NR	NR
Ennis et al. [16]	11.1	NR	NR
Tilki et al. [17]	1.0	8.8	15.7
Kishan et al. [19]	92.4	11.3	8.7
Westover et al. [29]	1.0	6	6

NR: not reported.

## Discussion

The present systematic review and meta-analyses assessed the comparative survival effectiveness of RT and RP as a definitive therapy with curative intent of PCa patients with high-risk features. Although we found that RT irrespective of RT type (i.e. EBRT alone or plus BT) resulted in significantly worse OS and CSS compared to RP, patients who underwent a EBRT plus BT combination had OS and CSS that were not inferior to RP. However, EBRT alone (i.e. without concomitant BT) was inferior to RP with regards to OS and CSS.

While there is no direct, well designed comparison of RP vs RT as the first step in a multimodal therapeutic concept in concordance with our meta-analyses, cumulative data support the concept of combining maximal RT consisting of EBRT plus BT with androgen-deprivation therapy (ADT) as the first-line multimodal strategy for therapy of high-risk PCa [10,12,16,17,19,29]. Indeed, major guidelines recommend ADT combined with EBRT plus BT, based on the OS benefits shown in several RCTs that compared it to EBRT plus ADT [35]. There is to date no comparison between the different multimodal therapies (i.e. EBRT plus BT and ADT) vs RP with RT vs RP with ADT, etc. Some single institution and small cohorts included in this systematic review tried to conduct a fair comparison between those strategies (i.e. RP plus adjuvant/salvage RT and ADT) [17,19,36,37]; however, the inherent selection bias limits any fair comparisons (Table 2), this is especially true for in multi-institutional and population-based datasets and registries, while they are at least likely to suffer from systematic bias based on their geographic and specialty representativeness.

Today, RT as adjuvant or salvage strategy is supported as a part of a multimodal therapy after RP by major guidelines for patients with high-risk PCa based on data recruiting from several RCTs [38–40]. However, more studies that compared EBRT plus BT to RP have not reported postoperative RT usage, therefore making fair comparisons impossible [9,10,12,14,16]. Limitations for optionally combining BT with EBRT include dose distribution and prostate size, reflecting a selection bias that may reflect the local tumour burden. In contrast to most cohort studies that assessed EBRT (i.e. without a concomitant BT) vs RP for high-risk disease, we found a significantly worse OS and CSS [9,11,18,22]. Finally, until well-designed RCTs assess survival outcomes between MaxRT and MaxRP, a multidisciplinary approach should be considered in treating patients with high-risk PCa beyond the results of survival outcomes of cohort studies.

The main limitation of the present systematic review and meta-analysis was the lack of well-designed controlled trials. However, because of this lack, we believe that this systematic review and meta-analysis might help frame and equipoise decisions to guide patients’ counselling as part of the shared decision process [5]. Another limitation was the heterogeneity across studies regarding the age of included patients, usage of ADT and its ADT duration, usage of adjuvant or salvage therapies such as postoperative RT after RP. Moreover, there was no precise data regarding the number of ADT patients, making subgroup analysis impossible. Indeed, designing a cohort study considering and adjusting for all variables’ effects is unlikely to be possible/to be performed. On the other hand, it is clear that the included multi-institutional cohorts suffer from a significant selection bias that limits the validity of the findings assessing from the former. Moreover, the effect of delayed definitive therapy due to the coronavirus disease 2019 (COVID-19) pandemic should be considered in future studies [41,42].

## Conclusions

According to this systematic review and meta-analyses, patients with high-risk PCa who received EBRT alone (without a concomitant BT) as a first-line definitive therapy had worse OS and CSS than those who underwent RP. However, EBRT plus BT as a multimodal RT was not inferior to RP in high-risk PCa. These data support the need for a multimodal strategy to achieve optimal therapy in high-risk PCa. RT as a primary definitive strategy could be better when combined with EBRT and BT to achieve maximal radiation dose combined with ADT. The role, timing, indication of postoperative RT after RP, and the type and duration of ADT for each patient need assessment and evidence. Until then, we would postulate based on the findings of our present study an equipoise of EBRT with BT for local control as a part of multimodal flexible and dynamic treatment strategy tailored to each tumour in each patient.

## Supplementary Material

Supplemental Material
