# Supplementary figures and images for: Radiation therapy compared to radical prostatectomy as first-line definitive therapy for patients with high-risk localised prostate cancer: An updated systematic review and meta-analysis
Source: Arab J Urol. 2022 Mar 30;20(2):71–80. doi: 10.1080/2090598X.2022.2026010 (PMC9067961; doi:10.1080/2090598X.2022.2026010)

| B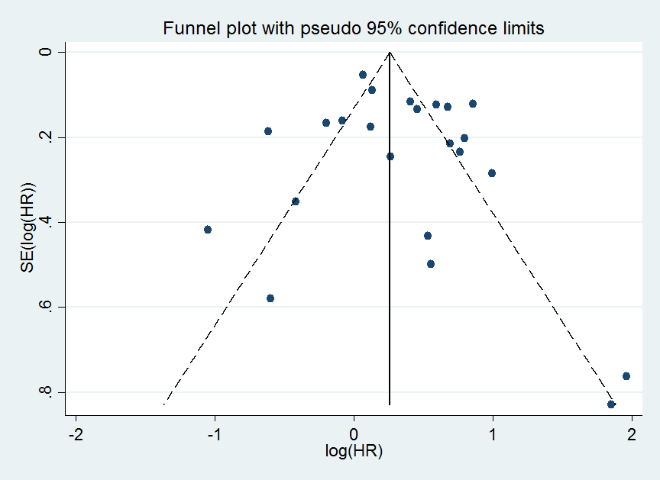 | A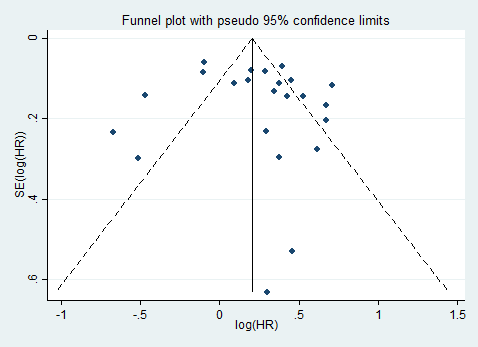 |
| --- | --- |
| 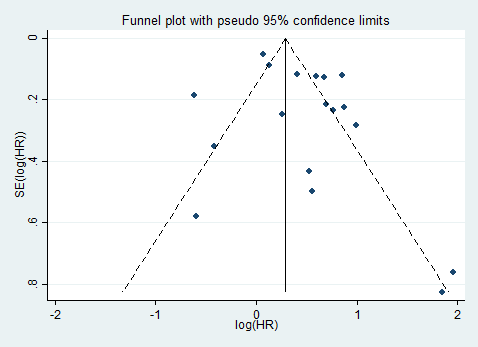D | C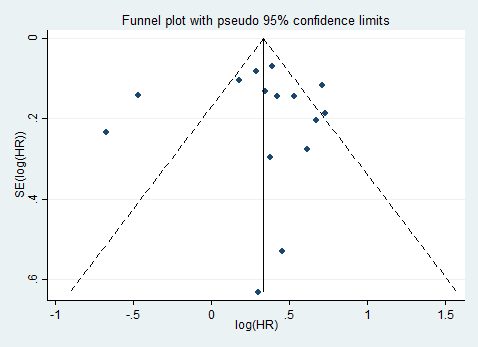 |
| 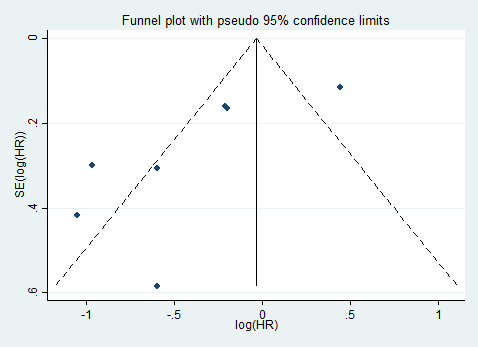F | 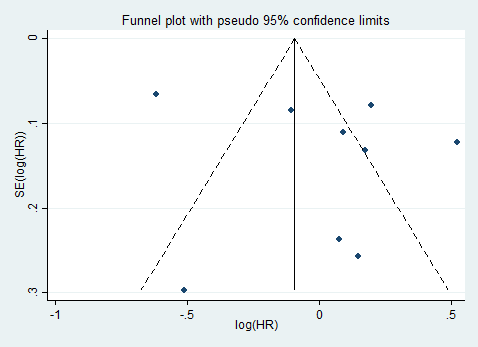  E |
| Figure 1S: Funnel plots of the six meta-analyses | |

Supplement: Supplemental Material [file TAJU_A_2026010_SM8498.docx]
